# Money Management in Multiple Sclerosis: The Role of Cognitive, Motor, and Affective Factors

**DOI:** 10.3389/fneur.2019.01128

**Published:** 2019-10-23

**Authors:** Goverover Yael, Chiaravalloti Nancy, DeLuca John

**Affiliations:** ^1^Department of Occupational Therapy, New York University, New York, NY, United States; ^2^Kessler Foundation, West Orange, NJ, United States; ^3^Department of Physical Medicine and Rehabilitation, New Jersey Medical School, Rutgers University, Newark, NJ, United States

**Keywords:** activities of daily life (ADL), multiple scleorsis (MS), money management, cognition, executive functions, quality of life

## Abstract

**Introduction:** Few studies have examined the motor, cognitive, and emotional factors involved in effective money management in persons with multiple sclerosis (MS). The aim of this study was to assess money management in persons MS and examine whether cognitive, motor, and emotional processes can predict money management.

**Methods:** This study included 72 persons with MS and 26 healthy controls (HC). Using an *a priori* definition of efficient vs. inefficient money management skills, based on the money management questionnaire (self and others), and performance on Actual Reality™ (AR) money management items, MS participants were divided into two groups: efficient or inefficient money management (MS Efficient- MM, *n* = 34 vs. MS Inefficient-MM, *n* = 38). These groups were compared on cognitive, motor, and emotional variables.

**Results:** Participants in the MS efficient MM group performed significantly better on executive function and processing speed measures, as well as performance on the 25WT. The MS Efficient -MM group also showed significantly less affective symptomatology (depressive and state anxiety). Importantly, HC performed similarly to the Efficient MM group on these tests. Good executive functioning and low depressive symptomatology predicted efficient money management.

**Conclusions:** This study characterizes some of the major problems and underlying impairments persons with MS are encountering in money management. Practitioners working with persons with MS should be aware that executive function impairments together with depressive symptomatology could signal possible money management dysfunction. The early identification of at-risk persons for money management difficulties could have a profound impact on the quality of life for this subsample of the MS population.

## Introduction

Multiple sclerosis (MS) is a common neurodegenerative disease affecting adults between the ages of 20 to 50, and is two to three times more common in women than men (1). The disease is characterized by inflammation, demyelination, and axonal loss, while chronic axonal degeneration succeeds later. The disease presents with a variety of symptoms including pain, fatigue, poor muscle control, balance and postural difficulties, cognitive impairments, and optic neuritis (2, 3). MS has a considerable impact on a patient's everyday functioning, quality of life, and the costs of disease management are substantial (4, 5).

Money management is a critical skill for everyday functional independence. The ability to perform tasks, such as managing cash, banking, paying bills, and budgeting, are all necessary for successful participation within the community (6). Although it had been already established that cognitive impairments are predictive of everyday functioning limitations [e.g., (7, 8)], an effort has been made on identifying cognitive abilities that are linked directly with specific real-world tasks, such as managing finances, to target intervention strategies (9, 10). Two studies (11, 12) were conducted specifically targeting money management with persons with MS. Both studies have shown that (1) participants with MS have more problems managing finances compared with healthy controls (HC) and (2) financial management tasks require several underlying cognitive abilities, such as working memory and, executive function (11, 12). This current study extends these prior studies (11, 12), by directly focusing on the financial management outcome of patients with MS as the primary objective, involving a larger sample of patients with MS than used in previous studies, and using collateral reports provided by participants' informants (e.g., caregiver, spouses, or siblings).

The primary aim of the present study was to first describe the main obstacles in money management of persons with MS with inefficient money management compared to persons with MS with efficient money management and HC. Second, we sought to examine the role of cognition, motor performance, and depressive symptomatology in predicting the functional outcome of money management in persons with MS. Our hypotheses were that (1) participants with MS with efficient money management will perform similarly to HC on cognitive tests, and report similar levels of affective symptomatology. Furthermore, HC and persons with MS with efficient money management will have better cognitive functioning and affective symptomatology than participants with MS with inefficient money management. (2) Impairment in executive functions, would predict group membership of efficient vs. inefficient money management in persons with MS after controlling for motor skills and affective symptomatology.

## Methods

### Participants

Participants consisted of 26 HC and 72 individuals with clinically definite MS [based on (13)] between the ages of 18 and 65 years. This study was designed with 0.80 probability of finding a significant difference between the groups. Based on comparison's effect size observed in the present study (*d* = 0.35), power analysis indicated that an n of ~90 would be needed to obtain statistical power at the recommended 0.80 level (14).

Participants with MS were recruited from support groups, advertisements, and from the Kessler Foundation. HC were recruited from advertisements and by word of mouth. All recruitment and study procedures were approved by the Institutional Review Board, consistent with the Health Insurance Portability and Accountability Act (HIPAA). Participants were excluded if they had any neurological or medical condition other than MS, had an exacerbation of symptoms and/or steroid treatment within the past month, had insufficient visual acuity to see the test materials, and did not speak English. Participants similarly could not participate in any cognitive rehabilitation program at the time of the study. Demographic characteristics are described in Table 1. Additionally, participants in the present study self-identified informants, who also consented to participate in this study. Ninety-eight Informants (26 of the HC and 72 of the persons with MS) were either a friend, relative, or a care taker and were identified by the study participants as someone who knows them very well and sees them regularly.

**Table 1:** Demographic characteristics of MS and HC.

	**MS inefficient (*n* = 38)**	**MS efficient (*n* = 34)**	**HC (*n* = 26)**	**Test**	***p***	**Tukey**
Age	50.1 ± 7.9	51.6 ± 9.3	44.4 ± 10.5	*F* = 4.8	0.01	c>b,a
Education	15.5 1.9	15.8 ± 1.9	17.2 ± 1.8	*F* = 6.1	<0.01	c>b,a
Gender
Male	21.1% (*n* = 8)	11.8% (*n* = 4)	34.6% (*n* = 9)	${\chi^{2}}_{\left(2\right)}$ = 3.8	0.14	
Female	78.9% (*n* = 30)	88.2% (*n* = 30)	65.4% (*n* = 17)			
Disease type						
Relapsing remitting	80%	95.7%	NA	${\chi^{2}}_{\left(2\right)}$ = 4.5	0.10	
Primary progressive	17.5%	0	NA			
Secondary progressive	2.5%	4.3%	NA			
Disease duration (month)	198.3 ± 121.9	198.4 ± 104.8	NA	*F* = 0.00	0.99	
MSFC-*z* score	−0.36 ± 0.68	−0.05 ± 0.56	0.17 ± 0.43	*F* = 6.5	<0.01	c>a; c>b
Employment (%)						
Disability/unemployed	63.2	47	11.5	χ^2^ = 48.1	<0.001	
Part-time work	18.4	20.6	15.4			
Student	0	0	11.5			
Volunteer	2.6	2.9	0			
Full-time work	15.8	20.6	61.5			

*MS, Multiple sclerosis; HC, healthy controls; MSFC, Multiple sclerosis functional composite*.

### Measures

#### Money Management

Overall money management status was assessed using two assessment methods: the performance based Actual Reality^TM^ (AR), and self- and informant ratings of the Money management questionnaire (described below).

##### Actual reality

Actual Reality^TM^ (AR) (8, 11) is a performance-based functional assessment that uses the internet to accomplish the actual real world task of purchasing a cookie bouquet from a business website. This task required participants to choose an appropriate cookie bouquet within a specified price range while taking into account the cost of shipping and handling. To score money management within AR, five behaviors within AR that are related to money management were targeted: staying within the indicated price range, using the credit card correctly, choosing an appropriate cookie bouquet, performing the task at an efficient pace and correctly responding to unforeseen occurrences [similar procedure described elsewhere (11)]. A score of a 0 (no error), 1 (minor error), or a 2 (major error) was given for performance of each of these behaviors indicated above. The scores depended on the significance and frequency of the errors made during performance. The score could range from 10 (severe deficit) to 0 (competent performance). AR has moderate to large Interrater Reliability, ranging from 0.79 to 0.89 and moderate test-retest reliabilities with intraclass correlations ranging from 0.5 to 0.83. AR also has good discriminant and concurrent validity for use with person with MS (15).

##### Self and informant-report money management

Money management was also assessed by a self-report and an informant report questionnaire (16) that was designed for patients with acquired brain injury and their informant. The questionnaire includes 11 short, concrete questions. On the patient form, the questions focus on whether the patient did or did not perform money management skills such as paying bills, using the ATM, budgeting, and borrowing money. Note that the patient is not asked to rate the quality or indicate any reasons for his/her performance. For example, one question asks, “Do you pay the rent late?” *Never (score of 0), Sometimes (score of 1)*, or *Often (score of 2)*. For the informant version, the form consists on the same questions as in the patients form. The scores for each form can range from 0 to 22, with a lower score indicating fewer problems managing money.

MS participants were divided into two groups based on money management abilities, as assessed with the AR money management portion (11) and the money management questionnaires (self and informant report). Scores of the both money management questionnaires and AR were summed and averaged for the participants with MS. Based on a median split of the summary and average scores, a score of 3 (observed range: 0.33–9) was *set* as cut-off to distinguish patients with efficient (score of 3 and lower; MS Efficient-MM) and inefficient (MS Inefficient -MM) (higher score than 3) money management. Note that all HC money management scores were lower than 3 except for one and thus was excluded from the analyses.

#### Affect Symptomatology

Depression and Anxiety were assessed using the Chicago Multi-scale Depression Inventory (CMDI) (17, 18) and the State and Trait Anxiety Inventory (STAI) (19), respectively. These questionnaires are based on self-report where participants are asked to rate their mood (i.e., depression and anxiety) on a 4 or 5-point Likert scale.

#### Cognitive Skills

Learning and memory: Verbal memory and learning were assessed by the Selective Reminding Test (SRT) (20). *Z*-score of the SRT was used in this study as dependent variables. Visual learning and memory were assessed by the Brief Visuospatial Memory Test-Revised (BVMT-R) (21). Total Recall across the three learning trials and the Delayed Recall t-scores served as the dependent variables.

Executive functions were assessed using the Delis-Kaplan Executive Function System (DKEFS) (22) letter-number sequencing trails subtest. The DKEFS scaled score (SS) was used in the analyses.

Processing speed and working memory were assessed using the (1) Symbol Digit Modalities Test SDMT; oral version (23); higher z-scores indicate faster processing speed and served as the dependent variable; and (2) Paced Auditory Serial Addition Test (PASAT) (24); There are two trials of 60 numbers each. The first consists on a 3-second inter-stimulus interval and the second on a 2-second inter-stimulus interval. Total number correct responses across the two trials served as the dependent variable.

#### Physical Functioning

Two subtests of the MS Functional Composite measure (25) were used: the Timed 25-Foot Walk Test (TWT) to assess lower limb function, and the 9-Hole Peg Test (9-HPT) to assess upper limb function. Z scores of these measures were calculated based on published norms (26, 27).

### Procedure

Potential participants were screened according to the inclusion/exclusion criteria described above during an initial phone conversation. All participants had to sign an informed consent form approved by the Institutional Review Board before study enrollment and then were scheduled for testing. During the testing, participants performed the AR task and the neuropsychological tests, and completed questionnaires to assess money management skills, and affective symptomology (order was randomized across subjects).

### Data Analysis

Group differences for demographics, cognitive performance, affect symptomatology, and physical performance were each analyzed by one-way analysis of variance with age and education as covariates (ANCOVA) with Tukey *post-hoc* analyses. For each item/question on the money management survey, responses were divided into two, with responses of 0 indicating “no problems” and responses of 1 and 2 indicating there were “problems” [based on (16)]. Multiple planned comparisons were analyzed using likelihood ratios to examine the individual items related to money management where individuals with MS (Efficient vs. Inefficient MM) were more likely to have problems compared to HC. These comparisons were also used to examine the AR task items. Lastly, a backward stepwise logistic regression with group membership (Efficient vs. Inefficient MM) as the criterion variable was used to investigate which of the independent variables could best predict efficient vs. inefficient MM functioning.

## Results

As shown in Table 1, there were significant differences between HC and MS in years of education [*F*_(2, 94)_ = 6.1, *p* < 0.01], disability score (MSFC) and employment status. The three groups (MS Efficient-MM, MS inefficient-MM functioning and HC) did not differ with respect to gender and age, but they differed on years of education, MSFC score and employment status. The two MS groups were more likely to be unemployed, and have less years of education and lower disability score compared with HC.

### Characteristics of the Difficulties With Money Management Across the Groups

Table 2 reports the problems with money management reported across the three groups based on the money management questionnaire. The MS inefficient-MM group reported problems with money management included using an ATM, paying the rent or bills late, owing money, spending all their money within the first few days of receiving it, going without essentials such as food because they had run out of money, impulse buying, and spending money on things they do not really need and needing to borrow money because they ran out of money. Similar patterns were reported by the informants of the participants, with the MS inefficient MM functioning group informants reporting similar frequencies of MM problems as the participants themselves.

**Table 2:** Percentage of participants in the MS inefficient and efficient MM functioning groups and HC group reporting problems in money management on the money management survey.

	**% MS inefficient (*n* = 38)**	**% MS efficient (*n* = 34)**	**% HC (*n* = 26)**	**Likelihood ratio**	***p***
Problems with ATM	21.2	9.1	0	9	0.01
Don't often check change	36.4	31.8	42.3	0.57	0.75
Pay bills or rent late	33.3	0	0	22.3	0.00
Thrown out of accommodation	9.1	0	0	5.5	0.06
Owe money for debts	42.4	0	0	29.6	0.00
Spend all money within first few days	39.4	4.5	3.8	16.7	0.00
Go without essentials	24.2	0	0	15.6	0.00
Problematic impulse buying	30.3	4.5	0	15.7	0.00
Spend all money on things they like	30.3	4.5	0	15.7	0.00
Need to borrow money	57.6	0	0	43.2	0.00

*ATM, automatic teller machine; MM, money management*.

In terms of money management on the AR task, the main difficulties presented by the MS inefficient MM group were that they committed more credit card errors, performed the task at a slower pace, did not choose the best option in terms of price, and did not respond efficiently to unexpected issues in comparison to the efficient MM group and HC groups (see Table 3).

**Table 3:** Percentage of participants in the MS inefficient and efficient MM and HC groups who performed more errors on the AR money management skills.

	**% MS inefficient (*n* = 38)**	**% MS efficient (*n* = 34)**	**% HC (*n* = 26)**	**Likelihood ratio**	***p***
Going over the price range	73.6	58.8	46.1	7.6	0.11
Credit card errors	42.2	35.3	15.4	9.4	0.05
Pace	76.3	50	26.9	24.1	<0.001
Choosing the best option	97.4	85.3	73	16.5	0.002
Noticing and responding to unexpected issues	90.5	55.8	34.6	33.0	<0.001

### Cognition, Motor Performance, and Depressive Symptomatology and Money Management

Comparisons between the three groups on cognitive and motor skills and affective symptomatology while controlling for age and education are presented in Table 4. On all cognitive measures except the PASAT, participants in the inefficient MM group performed significantly worse than participants in the efficient MM group, while HC performed similarly to the efficient MM group. On the BVMT delayed recall however, there were no significant differences between the inefficient MM group and the efficient MM group. The HC group performed significantly better than both MS groups.

**Table 4:** Difference between groups in cognitive, physical, and affective functioning while controlling for age and education as covariates.

	**MS inefficient**	**b. MS efficient**	**c. HC**	***F***	***p***	**Tukey**
**COGNITIVE**						
Memory						
BVMT-imm *t*-score	40 ± 14.3	45.4 ± 14.7	53.9 ± 7.5	5.8	<0.01	c>a
BVMT delayed	42.9 ± 14.4	47.9 ± 13.6	57.1 ± 4.7	7.6	<0.01	c>a c>b
SRT-*z* score	−2.1 ± 1.3	−1.2 ± 1.2	−0.85 ± 1.1	7.9	<0.01	c>a b>a
Processing speed						
SDMT *z* score	−1.02 ± 1.5	−0.19 ± 1.1	0.33 ±.72	7.5	<0.01	c>a b>a
PASAT 2 & 3	73.7 ± 22.7	80.5 ± 20.7	82.4 ± 21.9	0.93	0.39	None
Executive functions						
DKEFS Trails SS	8.6 ± 3.7	10.9 ± 3.3	11.2 ± 2.9	5.8	0.004	c>a b>a
**AFFECT SYMPTOMATOLOGY**						
CMDI-mood	50.3 ± 9.6	45.9 ± 7.9	45.6 ± 5.8	3.4	0.03	None
CMDI-evaluative	53.3 ± 13.7	45.9 ± 5.8	44.6 ± 1.4	7.8	<0.01	c>a b>a
CMDI-vegetative (Fatigue)	63.4 ± 13.2	53.3 ± 11.7	48.7 ± 7	11.7	<0.01	c>a b>a
STATE Anxiety	53.9 ± 10.6	44.6 ± 8.5	46.1 ± 9.5	8.9	<0.01	c>a b>a
TRAIT Anxiety	58 ± 12.4	47.8 ± 9.2	50.7 ± 11.3	7.5	<0.01	c>a b>a
**PHYSICAL**						
9-HPT *z* score	−0.53 ± 0.86	0.01 ± 0.76	0.57 ± 0.50	11.6	<0.01	c> a b>a
25 foot walk *z* score	0.13 ± 0.37	0.29 ± 0.13	0.40 ± 0.07	5.5	<0.01	c>a

With regard to affective symptomatology, the inefficient MM group reported significantly higher depressive and state anxiety symptomatology compared to efficient MM and HC. HC and MS-efficient MM reported similar symptomatology. A slightly different pattern was noted with regard to motor skills performance. On both the 25 FW and the 9HP MS-efficient MM performed similarly to the MS-inefficient group and the HC group. However, HC performed significantly better than inefficient MM, MS.

### Which Is the Best Predictor of Inefficient vs. Efficient Money Management in Patients With MS Only: Cognitive, Motor, or Affect?

A backward logistic regression analysis was performed to examine the relative contribution of cognitive, motor skills, and affect symptomatology in predicting efficient MM vs. inefficient MM functioning. Four predictors were included in this regression model, based on their significance in determining money management skills. These predictors were, executive functions score (DKEFS trails SS), processing speed score (SDMT *z* score), motor performance (9 HP *z* score) and depressive symptomatology (CMDI total t score). A test of the full model with backward stepwise method with the 4 predictors against a constant only model was statistically reliable [*X*^2^_(2, N = 70)_ = 19.7, *p* < 0.001], indicating that the predictors (CMDI: Odds ratio = 1.1; 95% CI = 1.03–1.2; *p* = 0.04; DKEFS: Odds ratio = 0.81; 95% CI = 0.68–0.97; *p* = 0.02) reliably distinguished between participants who had efficient MM vs. those who had inefficient MM (as described in Table 5). Overall, prediction success of the model was 67.1% and only executive functions and depressive symptomatology reliably predicted persons with MS with efficient vs. inefficient MM.

**Table 5:** Logistic Regression Analysis with cognitive, physical, and affective Measures as Predictors of Group Membership (efficient MM vs. inefficient MM).

**Measures**	**B**	**SE**	**Wald**	**df**	**sig**
**Step 1**					
CMDI	0.10	0.03	8.2	1	0.004
9HP	−0.30	0.49	0.38	1	0.53
SDMT	−0.06	0.34	0.02	1	0.87
DKEFS	−0.16	0.11	2.1	1	0.14
Constant	−3.5	2	3.1	1	0.07
**Step 2**					
CMDI	0.10	0.03	8.2	1	0.004
9HP	−0.29	0.45	0.40	1	0.52
DKEFS	−0.16	0.10	2.3	1	0.12
Constant	−3.6	1.9	3.3	1	0.07
**Step 3**					
CMDI	0.10	0.03	8.5	1	0.004
DKEFS	−0.20	0.09	5.3	1	0.02
**Constant**	−3.2	1.9	2.9	1	0.09

*CMDI, Chicago Multi-scale Depression Inventory; DKEFS, Delis-Kaplan Executive Function System; 9HP, 9-Hole Peg Test*.

## Discussion

The results of the current study show that persons with MS may struggle to perform fundamental money management tasks, which may have significant negative effects on their day to-day life. The main problems that were reported by persons with MS who have inefficient MM are owing money to others, the need to borrow money, and also spending money they have within a few days from the time they received it. During money management performance, problems in making appropriate choices related to price, choosing the most appropriate items and reviewing prices were observed. Thus, participants with MS in the inefficient-MS MM group clearly present with several money management errors/problems that can be extremely problematic to managing independent daily life. These problems can also lead to significant economic and safety consequences for patients and significant stress and burden for caregivers (28). It is important however, to note that the present study also showed that not all participants with MS have money managment issues; some individuals with MS have comparable abilities in money managment functioning to HC. It is thus important to determine who is at risk for the development of MM difficulties to avoid negative consequences.

A second goal of the study was to examine the underlying characteristics in persons with MS with money management difficulties, and examine the role of cognition, motor performance, and depressive symptomatology in predicting the functional outcome of money management in persons with MS. Results showed that the HC and efficient MS MM groups differed from the MS-inefficient MM group with respect to each of these aspects of functioning.

With regard to cognition, the MS-inefficient MM group performed worse on verbal memory, executive function and processing speed measures as compared with HC and the MS-efficient MM. Those with efficient MM skills performed similarly to HC on these tests. More specifically, participants in the inefficient MM group performed poorer on new learning and recall (SRT) than the comparison groups. This finding suggest that those patients with inefficient MM also have difficulties in learning and memory of verbal information. Consistent with this finding, impairment in verbal memory has been suggested to be a predictor of work impairment in persons with MS (29) and other clinical populations (30). In addition to differences in verbal memory, there were also significant differences noted between the groups with regard to executive functioning, as documented by the DKEFS letter-number sequencing subtest. MS participants with inefficient MM functioning scored significantly below HC. In considering the relationship between task and financial management, one must consider the task demands of effective financial management. That is, when managing finances, it is important to adjust to changes in income and expenses as well as to control spending. Indeed, these constructs are necessary for effective completion of the letter number sequencing task as well. That is, to successfully complete the task one must exhibit impulse/inhibitory control, similar to that which is necessary to curb unnecessary spending. In addition, the task requires mental flexibility/set shifting, such as that which may be needed when one must generate solutions to financial challenges and not perseverate on the manner in which one always managed income and expenditures. The final cognitive construct determined to be important to MM ability was *processing speed*. As can be seen in the literature, numerous studies have demonstrated the importance of processing speed in everyday functions [e.g., (5, 31)].

In terms of affective symptomatology, HC and participants with MS with efficient MM skills were less anxious and less depressed than those in the inefficient MM group. Those with efficient MM skills performed similarly to HC on these tests. We should note that all MS participants, across groups, showed mild symptoms of depression, and anxiety. Affective symptomatology may have a complex relationship with self-appraisal of personal abilities in MS (32). The absence of depression may be related to overestimation of abilities, while mild depression may be related to accurate self-assessment (33). It is also relevant to consider the role of coping in this relationship. That is, Arnett and Randolph (34) showed that patients with MS whose depressive symotomatology had worsened showed decreased active coping strategies. Although the nature of our design cannot determine causality, it may be possible that increases in depressed mood, may lead to decreased use of strategies which may affect every day functioning, including money management.

It is similarly important to note that individuals with inefficient money management skills also had worse motor performance on the 25-foot walk test compared to efficient MM and HC. This confluence of decline across measures of cognition, physical functioning, and money management skills may reflect an overall functional decline consistent with increased general disability.

Lastly, we hypothesized that executive functions would predict MM quality above and beyond affective symptomatology and motor skills. This hypothesis was partially confirmed because both executive function and depressive symptomatology were significant predictors of MM functional level. The results related to executive functions accord well with prior investigations in MS (11, 12). Prior research however, did not find that depressive symptomology is associated with MM. As such, depressive symptomatology may serve as a key vulnerability for MM among patients with MS. We concur with the Tracy et al. (12) recommendation that future research should examine whether more specific aspects of executive function and depressive symptomatology contribute to inefficient MM in MS.

The current research has a number of weaknesses. Participants were recruited through community-based lists and support groups. As a result, many participants in the study were independent in their daily life. Before generalizing the findings to all individuals with MS, further studies using participants with MS with a wider variety of disability could assist in determining the relationship between competence to manage personal finances and cognitive status. There are a limited number of performance-based measures to assess MM in clinical populations. In this study, we used the Money management questionnaire (self and informant reports) and few items from AR related to money management. Psychometric properties of both must be established before it can confidently be used by future studies. Lastly, it would be interesting to assess MM in an objective context. This will help clinicians assess it empirically without evoking subjective biases and errors.

## Conclusion

For individuals with MS and their families, MM may be a crucial activity of daily living. Impairment in MM can have clinical, psychological, economic, and legal implications (35). Therefore, practitioners working with persons with MS should be aware that cognitive impairment generally, and impairments in executive functions specifically, could signal possible MM limitations, and prompt the clinician to urge patients and families to engage in advance financial and legal planning. Furthermore, timely documentation and assessment of MM limitations can often prompt beneficial financial planning that could improve the economic, psychological, and legal implication of financial dysfunction in people with MS. Further research is needed to establish standardization and guideline for such issue.

## Data Availability Statement

The datasets generated for this study are available on request to the corresponding author.

## Ethics Statement

The studies involving human participants were reviewed and approved by Kessler Research Foundation New York University. The patients/participants provided their written informed consent to participate in this study.

## Author Contributions

GY conceptualized the study idea, drafted the paper, and did all the analyses and interpretation of data. DJ and CN revised it and added important intellectual content.

### Conflict of Interest

The authors declare that the research was conducted in the absence of any commercial or financial relationships that could be construed as a potential conflict of interest.

## References

[1] Tullman MJ Overview of the epidemiology, diagnosis, and disease progression associated with multiple sclerosis. Am J Manag Care. (2013) 19:S15–20. 23544716

[2] Goverover Y Genova HM DeLuca J Chiaravalloti ND Impact of multiple sclerosis on daily life. In: Chiaravalloti N Goverover Y editors. Changes in the Brain. New York, NY: Springer (2017). p. 145–165. 10.1007/978-0-387-98188-8_7

[3] Tappenden P McCabe C Chilcott J Simpson E Nixon R Madan J Cost-effectiveness of disease-modifying therapies in the management of multiple sclerosis for the medicare population. Value Health. (2009) 12:657–65. 10.1111/j.1524-4733.2008.00485.x 19508662

[4] Fredrikson S Economic impact of cognitive impairments in multiple sclerosis. In: DeLuca J Sandroff BM, editors. Cognition and Behavior in Multiple Sclerosis. Washington DC: American Psychological Association (2018). p. 207–22. 10.1037/0000097-011

[5] Kavaliunas A Danylaite Karrenbauer V Gyllensten H Manouchehrinia A Glaser A Olsson T Cognitive function is a major determinant of income among multiple sclerosis patients in Sweden acting independently from physical disability. Multiple Scler J. (2019) 25:104–12. 10.1177/1352458517740212 29143553

[6] Engel L Bar Y Beaton DE Green RE Dawson DR Identifying instruments to quantify financial management skills in adults with acquired cognitive impairments. J Clin Exp Neuropsychol. (2016) 38:76–95. 10.1080/13803395.2015.1087468 26595230

[7] Goverover Y Genova H Hillary F DeLuca J The relationship between neuropsychological measures and the timed instrumental activities of daily living task in multiple sclerosis. Multiple Scler J. (2007) 13:636–44. 10.1177/1352458506072984 17548444

[8] Goverover Y O'Brien AR Moore NB DeLuca J Actual reality: a new approach to functional assessment in persons with multiple sclerosis. Arch Phys Med Rehabil. (2010) 91:252–60. 10.1016/j.apmr.2009.09.022 20159130

[9] Harvey PD Stone L Lowenstein D Czaja SJ Heaton RK Twamley EW The convergence between self-reports and observer ratings of financial skills and direct assessment of financial capabilities in patients with schizophrenia: more detail is not always better. Schizophr Res. (2013) 147:86–90. 10.1016/j.schres.2013.02.018 23537475 PMC3650121

[10] Patterson TL Mausbach BT Measurement of functional capacity: a new approach to understanding functional differences and real-world behavioral adaptation in those with mental illness. Ann Rev Clin Psychol. (2010) 6:139–54. 10.1146/annurev.clinpsy.121208.131339 20334554 PMC3160788

[11] Goverover Y Haas S DeLuca J Money management activities in persons with multiple sclerosis. Arch Phys Med Rehabil. (2016) 97:1901–7. 10.1016/j.apmr.2016.05.003 27240432

[12] Tracy VL Basso MR Marson DC Combs DR Whiteside DM Capacity for financial decision making in multiple sclerosis. J Clin Exp Neuropsychol. (2016) 39:1–12. 10.1080/13803395.2016.1201050 27430343 PMC5224702

[13] Polman CH Reingold SC Banwell B Clanet M Cohen JA Filippi M Diagnostic criteria for multiple sclerosis: 2010 revisions to the McDonald criteria. Anna Neurol. (2011) 69:292–302. 10.1002/ana.22366 PMC3084507 21387374

[14] Cohen J Statistical Power Analysis for the Behavioral Sciences. New York, NY: Routledge (2013).

[15] Goverover Y DeLuca J Assessing everyday life functional activity using actual reality TM in persons with MS. Rehabil Psychol. (2018) 63:276–85. 10.1037/rep0000212 29878832

[16] Hoskin KM Jackson M Crowe SF Money management after acquired brain dysfunction: the validity of neuropsychological assessment. Rehabil Psychol. (2005) 50:355–65. 10.1037/0090-5550.50.4.355

[17] Chang C-H Nyenhuis DL Cella D Luchetta T Dineen K Reder AT Psychometric evaluation of the Chicago multiscale depression inventory in multiple sclerosis patients. Multiple Scler. (2003) 9:160–70. 10.1191/1352458503ms885oa 12708812

[18] Nyenhuis DL Luchetta T Yamamoto C Terrien A Bernardin L Rao SM The development, standardization, and initial validation of the Chicago multiscale depression inventory. J Personal Assess. (1998) 70:386–401. 10.1207/s15327752jpa7002_14 9697337

[19] Spielberger CD Gorsuch RL Lushene PR Vagg PR Jacobs AG Manual for the State-Trait Anxiety Inventory. Palo Alto, CA: Consulting Psychologists Press (1983).

[20] Buschke H Selective reminding for analysis of memory and learning. J Verbal Learn Verbal Behav. (1973) 12:543–50. 10.1016/S0022-5371(73)80034-9

[21] Benedict RHB Revision of the brief visuospatial memory test - revised. Psychol Assess. (1997) 8:145–53. 10.1037/1040-3590.8.2.145

[22] Delis D Kaplan E Kramer J Delis-Kaplan executive function system (D-KEFS). Can J School Psychol. (2001) 20:117–28. 10.1177/0829573506295469

[23] Smith A Symbol Digit Modalities Test (SDMT) Manual (Revised). Los Angeles, CA: Western Psychological Services (1982).

[24] Rudick R Antel J Confavreux C Cutter G Ellison G Fischer J Recommendations from the national multiple sclerosis society clinical outcomes assessment task force. Ann Neurol. (1997) 42:379–82. 10.1002/ana.410420318 9307263

[25] Fischer JS Rudick RA Cutter GR Reingold SC The multiple sclerosis functional composite measure (MSFC): an integrated approach to MS clinical outcome assessment. National MS Society Clinical Outcomes Assessment Task Force. Mult Scler. (1999) 5:244–50. 10.1177/135245859900500409 10467383

[26] Grice KO Vogel KA Le V Mitchell A Muniz S Vollmer MA *Adult* norms for a commercially available nine hole peg test for finger d*exterity*. Am J Occupat Ther. (2003) 57:570–3. 10.5014/ajot.57.5.570 14527120

[27] Kragt JJ Van Der Linden F Nielsen JM Uitdehaag BMJ Polman CH Clinical impact of 20% worsening on timed 25-foot walk and 9-hole Peg test in multiple sclerosis. Mult Scler. (2006) 12:594–8. 10.1177/1352458506070768 17086905

[28] Moberg PJ Rick JH Decision-making capacity and competency in the elderly: a clinical and neuropsychological perspective. NeuroRehabilitation. (2008) 23:403–13. 18957727

[29] Clemens L Langdon D (2018,. November). How does cognition relate to employment in multiple Sclerosis? A systematic Review. Mult Scler Relat Disord. 26:183–91. 10.1016/j.msard.2018.09.018 30268039

[30] Fujino H Sumiyoshi C Sumiyoshi T Yasuda Y Yamamori H Ohi K Predicting employment status and subjective quality of life in patients with schizophrenia. Schizophr Res. (2016) 3:20–5. 10.1016/j.scog.2015.10.005 PMC5506698 28740804

[31] Goverover Y Strober LB Chiaravalloti N DeLuca J Factors that moderate activity limitation and participation restriction in people with multiple sclerosis. Am J Occupat Ther. (2015) 69:1–9. 10.5014/ajot.2015.014332 26122682

[32] Goverover Y Kalmar J Gaudino-Goering E Shawaryn M Moore NB Halper J The relation between subjective and objective measures of everyday life activities in persons with multiple sclerosis. Arch Phys Med Rehabil. (2005) 86:2303–8. 10.1016/j.apmr.2005.05.016 16344027

[33] Harvey PD Twamley EW Pinkham AE Depp CA Patterson TL Depression in schizophrenia: associations with cognition, functional capacity, everyday functioning, and self-assessment. Schizophr Bull. (2017) 43:575–82. 10.1093/schbul/sbw103 27440672 PMC5463852

[34] Arnett P Randolph JJ Longitudinal course of depression symptoms in multiple sclerosis. J Neurol Neurosurg Psychiatr. (2006) 77:606–10. 10.1136/jnnp.2004.047712 PMC2117462 16614019

[35] Widera E Steenpass V Marson D Sudore R Finances in the older patient with cognitive impairment. JAMA. (2011) 305:698–706. 10.1001/jama.2011.164 21325186 PMC3799787

